# Experiences, Attitudes and Preferences of Postpartum Cisgender Women for HIV Prevention and Pre-Exposure Prophylaxis Education during Routine Postpartum Care

**DOI:** 10.1007/s10461-025-04663-5

**Published:** 2025-03-10

**Authors:** Caroline E. Mullis, Jessica McWalters, Alison J. Goldberg, Aloke Maity, Karina Avila, Sarit A. Golub, Marla J. Keller

**Affiliations:** 1 Department of Medicine, Albert Einstein College of Medicine and Montefiore Medical Center, 1300 Morris Park Ave., Bronx, NY 10461 USA; 2 Department of Obstetrics & Gynecology and Women’s Health, Albert Einstein College of Medicine and Montefiore Medical Center, 1300 Morris Park Ave., Bronx, NY 10461 USA; 3 Department of Psychology, Hunter College of the City University of New York, 695 Park Avenue, New York, NY 10065 USA; 4 Graduate Center of the City University of New York, 365 5th Ave, New York, NY 10016 USA

**Keywords:** HIV prevention, PrEP, Postpartum, Cisgender women

## Abstract

The postpartum period presents an opportunity to deliver comprehensive sexual health education. Understanding preferences of postpartum cisgender women (CGW) towards integration of HIV prevention education into postpartum care is necessary to support acceptability, appropriateness, and feasibility. Postpartum CGW were recruited during their postpartum hospital stay in Bronx, NY. Participants completed a survey including two best-worst scaling exercises that elicited preferences for receiving HIV prevention information and PrEP messaging. Utility scores were estimated using a hierarchical Bayes multinomial logit model and mathematically transformed into a probability scale presented as preference scores (PS), with higher numbers representing greater preference. Among 259 participants, 53% had never heard of PrEP before. Receiving information from a doctor was most preferred (13.4, 95%CI 12.7–14.0) followed by receiving information about HIV prevention combined with taking care of their health after having a baby (11.6, 95% CI 10.9–12.3). Latent class analysis revealed three classes: participants with greater preference for high touch services (e.g., in-person visits, doctor providing information), integrated information (e.g., HIV prevention information given in combination with sexual or maternal health information) and high technology services (e.g., a mobile phone application, telehealth visit). Participants preferred PrEP messaging related to themes of effectiveness, motherhood, safety and autonomy. Latent class analysis of PrEP messaging revealed two classes: one with participants preferring messages related to motherhood and safety and another preferring messages related to control and autonomy. The heterogeneity in preferences for receiving information at the patient level suggests a need for patient-centered, differentiated service delivery to support maximum receptivity.

**Supplementary Information:**

The online version contains supplementary material available at 10.1007/s10461-025-04663-5.

## Introduction

Despite more than a decade of availability of pre-exposure prophylaxis (PrEP) as an effective, user-controlled HIV prevention strategy, PrEP remains underutilized among cisgender women (CGW) [1–3]. While women represent 19% of new HIV diagnoses in the United States (US), they comprise only 8% of PrEP users [4]. Additionally, structural inequalities and systemic racism are evident in racial and ethnic health disparities in PrEP access in the US [5, 6]. While most new HIV diagnoses among US CGW occur in Black and Hispanic CGW, rates of PrEP use are lower in these populations [4, 6]. In New York City (NYC), racial and ethnic inequities in new HIV diagnoses persist in CGW [7]. Black and Latina/Hispanic women represent 50% and 34% of new HIV diagnoses respectively. However, in a survey of sexually active Black and Latina cisgender women in NYC, only 45% of participants in 2018 had awareness of PrEP and only 3% had ever used PrEP despite 30% responding that they were interested in taking PrEP [1]. Effective and equitable delivery of HIV prevention and PrEP education and services to CGW is crucial to support PrEP uptake and efforts to decrease rates of new HIV diagnoses.

The postpartum period presents a unique opportunity for delivery of comprehensive sexual health services, including HIV prevention education, PrEP and contraception [8]. The postpartum period is increasingly being recognized as an important period for supporting the health of CGW as many reproductive age women may not otherwise engage in routine preventative healthcare [9, 10]. The American College of Obstetrics and Gynecology guidelines for postpartum care recognize the need to support postpartum mothers in continued engagement in routine, well-woman care [9]. Prior qualitative work in Washington DC and NYC has demonstrated that despite women being interested in learning about HIV prevention strategies and PrEP during prenatal and postpartum care, these discussions rarely occur [8, 11].

Patient, provider and health system level barriers to delivery of HIV prevention and PrEP services must be overcome to support effective and equitable delivery of HIV prevention services to CGW in the postpartum period [12]. To effectively utilize the postpartum period to increase awareness of and engage CGW in HIV prevention services, identification of implementation strategies is crucial to maximize receptivity. This study aims to understand postpartum CGW’s (1) knowledge and attitudes about HIV prevention strategies, (2) preferences for receiving HIV prevention education as a part of postpartum care, and (3) preferences for messaging related to PrEP use.

## Methods

### Study Population

Patients were approached for participation by an Infectious Diseases physician (C.E.M.) or a Women’s Health nurse practitioner (J.M.) on a single inpatient unit during their postpartum hospital stay at the Jack D. Weiler Hospital of the Montefiore Medical Center in Bronx, NY. To be eligible for the study, participants had to be 18 years or older, postpartum, a cisgender woman (sex at birth female and gender identity of woman), self-reported without HIV diagnosis and able to complete the survey in English or Spanish. To evaluate representativeness of the study population, demographics of all postpartum women on the same inpatient unit at Weiler Hospital between January 1, 2024 and May 31, 2024 were obtained from the electronic health record using structured query language from Epic’s Clarity database.

### Survey Content and Design

Participants were surveyed about their experiences with and preferences for delivery of HIV prevention services. All participants were presented with the following definition and background on PrEP: “PrEP or pre-exposure prophylaxis is a medicine women can take to prevent getting HIV. When taken as prescribed, PrEP is highly effective and can reduce the risk of getting HIV from sex by about 99%”. This cross-sectional survey initially asked about prior knowledge of PrEP and attitudes towards experiences and services related to HIV prevention including PrEP. Participants were asked how strongly they agreed or disagreed with statements, such as “HIV prevention is important to me,” on a five-point Likert scale. Participants either agreeing or strongly agreeing were combined for analysis and compared to all other participants (those responding as strongly disagreeing, disagreeing, or neutral).

**Fig. 1:**
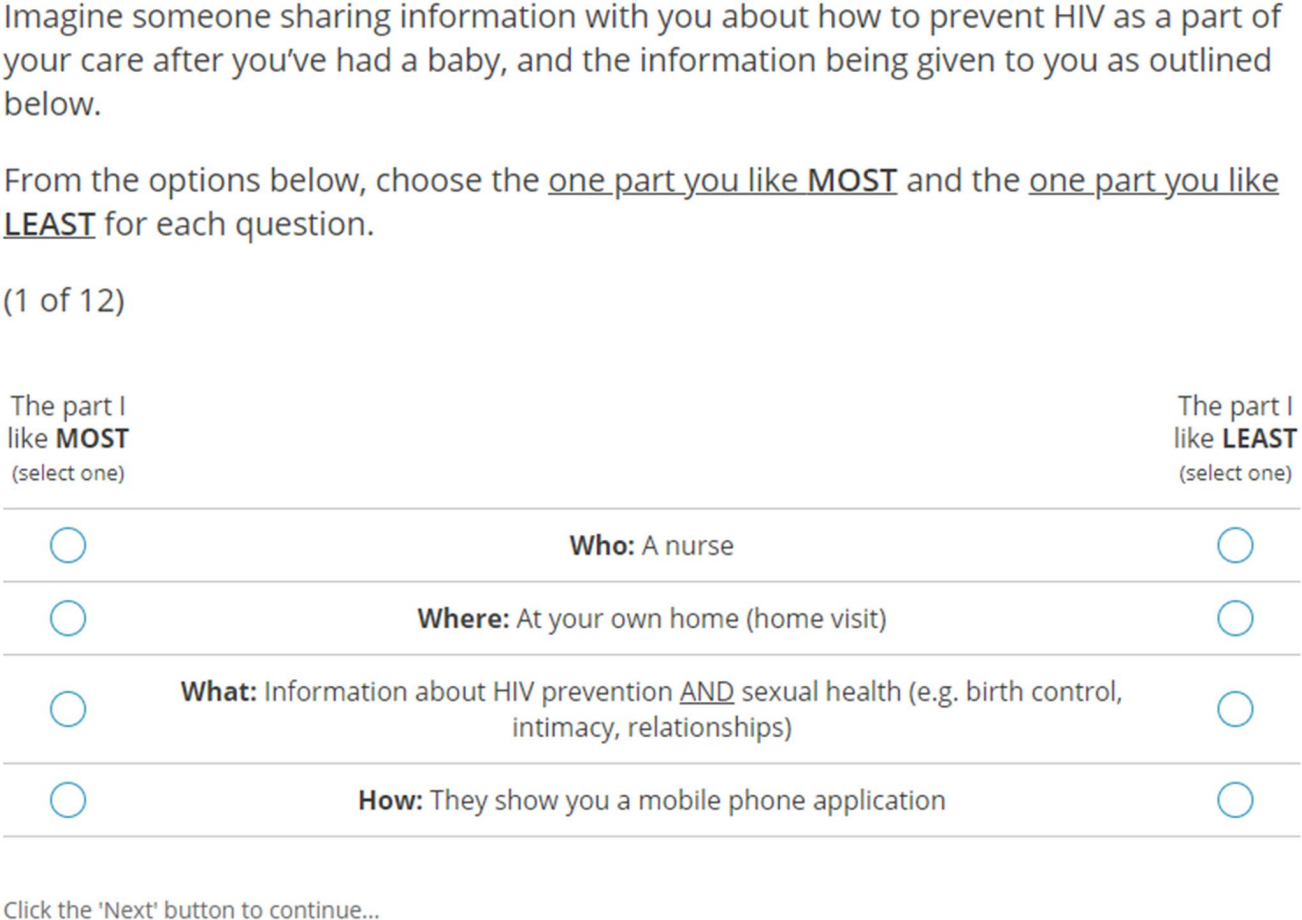
Example profile question for best-worst scaling exercise to elicit preferences for receiving HIV prevention information

The survey had two separate best-worst scaling (BWS) exercises, which are used to determine preferences by asking respondents to select the best and worst from a set of provided attributes or items [13]. The first was a BWS type two design (profile case) and assessed preferences about delivery of HIV prevention information during routine postpartum care. Preferences for four attribute categories were assessed, each with three or four levels: (1) who would provide the information (doctor, nurse, or counselor); (2) where would the information be provided (hospital, clinic, home, or telehealth visit); (3) what information would be provided (information about HIV prevention alone, information about HIV prevention and sexual health, information about HIV prevention and taking care of your health after you’ve had a baby); (4) how information would be provided (mobile phone application, website, talking, pamphlet). As a BWS type two design, each question in this exercise showed respondents a level of each attribute in a profile format and requested participants choose the part they liked most (best) and the part they liked least (worst) (see Fig. 1 for sample question).

The second BWS exercise was a type one design (object case) to elicit preferences about PrEP messaging and presented different attributes than the first BWS exercise [13]. Messages related to PrEP were created based on themes identified as facilitators for PrEP use among postpartum women in prior qualitative work [8]. Each participant was presented with four potential messages related to PrEP and asked to select which message they liked best and which message they liked least (a full list of messages is provided in the Results section, "Preferences for PrEP Messaging" ). BWS exercises were designed in Lighthouse Studio with MaxDiff Designer, which utilizes a programming-based algorithm to choose experimental designs with the best balance both in the number of times each item occurs and the number of times each pair of items occurs within sets. Demographics were asked at the conclusion of the survey.

Survey question language was simplified with the assistance of ChatGPT (OpenAI, 2023). The survey was piloted in ten individuals and modified to improve question clarity. The survey was available in English and Spanish. Participants completed the survey in the presence of research staff to ensure that participants were able to complete the survey in a safe environment and so that clarification could be provided if needed. Median completion time was 15.5 min (IQR 12.0-20.1). At the completion of the survey, participants were provided with additional information about PrEP, including where to access services. Participants received a $25 Amazon gift card for their participation.

### Sample Size and Statistical Analysis

A sample size of 250 participants was targeted based on a high precision (margin of error delta = 0.1) “rule of thumb” formula estimating 233 or 244 participants for BWS exercises one and two respectively [14].

Survey participant characteristics were summarized as mean or number and percentages. For each BWS exercise, respondent-level utility scores were estimated using a hierarchical Bayesian multinomial logit model and were rescaled into preference scores separately. Preference scores are defined as the likelihood of an item being chosen from a set of items with higher preference scores representing greater relative importance or preference for attributes. Preference scores are re-scaled for each exercise, sum to 100 and are ratio-scaled, meaning that an attribute with a score of 10 is twice as preferred or important to participants as an item with a score of five [15]. Preference scores allow only for relative interpretation. While preference scores are proportional to the probability of a participant choosing an item as best and not worst, they do not represent this probability. Evaluation of population preference scores, excluding participants with completion time in the fastest 2.5% and/or with individual fit-scores < 0.336 based on Sawtooth Software guidelines for identifying bad respondents [16], was performed as a sensitivity analysis. Latent class analysis was performed on both BWS exercises independently to characterize any potential preference heterogeneity. Separate latent class analyses were performed, one for each exercise, as each BWS exercise presented different attributes. Two to five class selections were explored and selected based on model fit, which was assessed using common indices (log-likelihood, Akaike’s information criterion, Bayesian information criterion, chi-square), as well as clinical relevance and interpretability of results. Total Unduplicated Reach and Frequency (TURF) analysis was conducted as an optimization approach to identify subsets of attributes that would reach the maximum number of respondents [17]. For BWS exercise one TURF analysis, portfolios containing four items were generated by applying prohibitions to evaluate one level per attribute (i.e., one who, one where, one what, one how). For BWS exercise two TURF analysis, two and three attribute combinations were evaluated without application of prohibitions. Reach is computed as the probability that the respondents will choose at least one of the items in the portfolio instead of other items of average utility. These analyses were performed in Lighthouse Studio 9.15.14 and the associated MaxDiff Analyzer (Sawtooth Software, Provo, UT).

Differences between latent classes were evaluated in bivariate analysis utilizing Chi-squared or Fisher’s exact tests for categorical variables and Wilcoxon rank sum tests for continuous variables. Two-sided p-values less than 0.05 were considered statistically significant. Bivariate analysis was performed on STATA (version 17.0, StataCorp, College Station, TX). Figures were generated using GraphPad Prism (GraphPad Software, Boston, MA).

### Ethical Review

This study was approved by the Institutional Review Board at the Albert Einstein College of Medicine (IRB # 2023–15249) and the City University of New York (IRB # 2022-0509-Hunter). All participants provided informed consent electronically prior to participation.

## Results

### Study Population

A total of 260 participants completed the survey between January 10, 2024 and May 17, 2024. One participant’s data were excluded because study staff observed another person helping the participant complete the survey. A total of 259 participants were included in the analysis. The mean age of participants was 30 years with age of participants ranging from 19 to 46 years (Table 1). Forty-six percent (109/238) of participants self-identified as Black and 60% (154/258) as Hispanic. Thirty-six participants (14%) completed the survey in Spanish. 56% (144/259) of participant’s highest level of education was a high school degree or less, and 74% (191/259) had Medicaid as their primary insurance prior to pregnancy. When comparing demographics of the survey participants to the patients admitted to the postpartum unit during the study period, there were no statistically significant differences in age, ethnicity and primary language (Supplemental Table 1). However, the survey population had a higher percentage of individuals identifying as Black (46% vs. 32%) or White race (17% vs. 7%) and lower percentage of individuals identifying as other race (30% vs. 55%) (*p* < 0.001). 

**Table 1:** Characteristics of participants completing survey (*n* = 259)

Characteristic	*n* (%)
Age in years- mean (range)^a^	30 (19–46)
Race^a^	
Black	109 (46%)
White	41 (17%)
Asian	17 (7%)
Other	71 (30%)
Ethnicity^a^	
Hispanic/Latina	154 (60%)
Non-Hispanic/Latina	104 (40%)
Primary Language	
English	223 (86%)
Spanish	36 (14%)
Highest Level of Education	
High School Degree or less	144 (56%)
Technical/Associate Degree or more	115 (44%)
Employment Status^a^	
Employed	149 (60%)
Not employed	99 (40%)
Insurance prior to pregnancy	
Medicaid	191 (74%)
Private health plan	52 (20%)
Other government plan	12 (5%)
Uninsured	4 (1%)
Relationship Status	
Single, not dating anyone	39 (15%)
Dating or talking to one or more people	11 (4%)
In a committed relationship	205 (79%)
Other	4 (2%)

^a^ Denotes missing data. Data presented includes age in 255 participants, race in 238, ethnicity in 258, employment status in 258

Most participants had never heard of PrEP before (53%) and among the 47% who had heard of PrEP only 4% of participants reported knowing a lot about PrEP (Table 2). Among those who were aware of PrEP, the most common sources of PrEP knowledge were commercials (44%), a doctor or nurse (36%), the internet (26%) and/or friends or family (21%). A higher percentage of participants who had prior knowledge of PrEP had accessed HIV or STI services prior to pregnancy relative to those who had not (43% vs. 28%, *p* = 0.01). Sixty-two percent of participants were interested in learning more about PrEP, and among the 38% who were not interested in learning more about PrEP, 68% (67/99) had heard of PrEP previously. When asked about their likelihood to use PrEP in the future, 27% responded that they “might” and 18% responded that they “would”. Fifty-four percent (140/259) of participants accessed HIV or STI services only during pregnancy, and 35% (90/259) of participants reported access to HIV or STI services prior to pregnancy.

**Table 2:** Characteristics of groups in latent class analysis of preferences for HIV prevention information delivery and Prep messaging

Characteristic (number responding to question)	Total *n* (%)	Latent classes for preferences of HIV prevention information delivery				Latent classes for preferences of PrEP messaging		
		Class 1 (*n* = 123)	Class 2 (*n* = 91)	Class 3 (*n* = 45)	*p*	Class 1 (*n* = 184)	Class 2 (*n* = 75)	*p*
Prior knowledge of PrEP (*n* = 258)					0.53			0.82
No	137 (53%)	68 (56%)	44 (48%)	25 (56%)		98 (54%)	39 (52%)	
Yes	121 (47%)	54 (44%)	47 (52%)	20 (44%)		85 (46%)	36 (48%)	
Interest in learning more about PrEP (*n* = 259)					0.95			0.71
Not interested	99 (38%)	46 (37%)	36 (40%)	17 (38%)		69 (38%)	30 (40%)	
Interested	160 (62%)	77 (63%)	55 (60%)	28 (62%)		115 (62%)	45 (60%)	
Likelihood to use PrEP in the future (*n* = 258)					0.12			0.52
Would not	142 (55%)	75 (61%)	48 (53%)	19 (43%)		99 (54%)	43 (58%)	
Might	70 (27%)	33 (27%)	22 (24%)	15 (34%)		49 (26%)	21 (28%)	
Would	46 (18%)	15 (12%)	21 (23%)	10 (22%)		36 (20%)	10 (14%)	
Prior access to HIV/STI services (*n* = 259)					< 0.05*			< 0.01*
Accessed prior to pregnancy	90 (35%)	35 (29%)	33 (36%)	22 (49%)		54 (29%)	36 (48%)	
Did not access prior to pregnancy	169 (65%)	88 (71%)	58 (64%)	23 (51%)		130 (71%)	39 (52%)	
Agree with the statement								
HIV prevention is important to me (*n* = 258)	229 (92%)	110 (90%)	82 (90%)	37 (82%)	0.31	163 (89%)	66 (88%)	0.80
I worry about getting HIV (*n* = 253)	103 (41%)	50 (42%)	43 (47%)	10 (24%)	0.04*	79 (44%)	24 (32%)	0.08
Preventing HIV and STIs are a priority for my health (*n* = 257)	223 (87%)	109 (89%)	78 (87%)	36 (80%)	0.29	157 (86%)	66 (89%)	0.47
I feel comfortable talking about PrEP with my provider (*n* = 257)	187 (73%)	91 (74%)	67 (74%)	29 (67%)	0.69	128 (70%)	59 (81%)	0.07
It would be helpful to have a way for partner(s) to get tested for HIV (*n* = 257)	178 (69%)	84 (69%)	68 (76%)	26 (58%)	0.11	123 (68%)	55 (73%)	0.36
It’s easy for me to get HIV and STI prevention services (*n* = 256)	194 (76%)	92 (76%)	75 (82%)	27 (61%)	0.03*	134 (74%)	60 (81%)	0.21
I want to learn more about ways to prevent HIV (*n* = 256)	146 (57%)	72 (59%)	57 (63%)	17 (40%)	0.03*	108 (59%)	38 (51%)	0.24
Creating a plan for my sexual health is useful (*n* = 255)	181 (71%)	86 (71%)	69 (76%)	26 (61%)	0.19	133 (74%)	48 (65%)	0.17
Creating a plan for my sexual health is overwhelming (*n* = 257)	54 (21%)	28 (23%)	17 (19%)	9 (20%)	0.75	46 (25%)	8 (11%)	0.01*

Abbreviations: PrEP- pre-exposure prophylaxis; HIV- Human immunodeficiency virus STI- sexually transmitted infection

*Denotes statistical significance with p-value < 0.05

### Preferences for Receiving Information About HIV Prevention as a Part of Postpartum Care

**Fig. 2:**
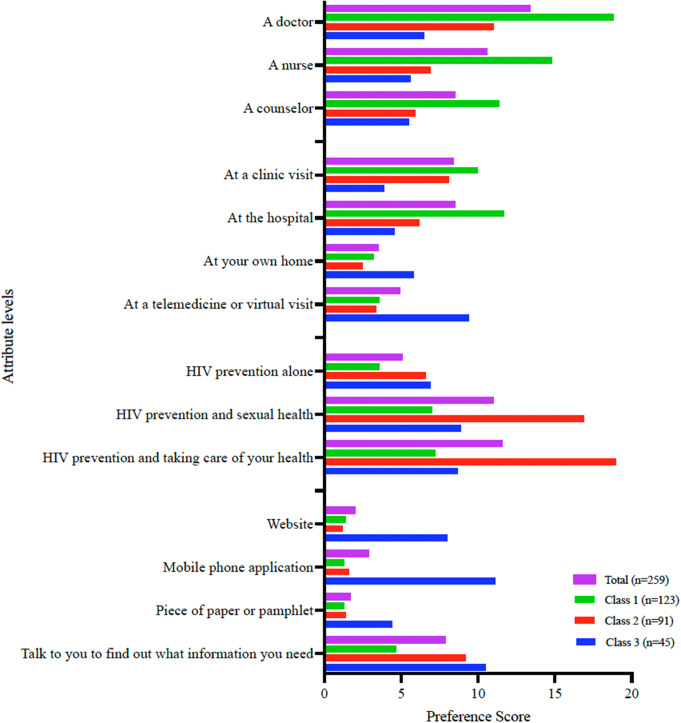
Preference scores from best-worst scaling exercise for delivery of information about HIV prevention as a part of postpartum care by total study population (*n* = 259), and three latent class groups

Among all survey participants, receiving information from a doctor was the most preferred attribute level (13.4, 95% CI 12.7–14.0) followed by receiving information about HIV prevention combined with taking care of their health after having a baby (11.6, 95% CI 10.9–12.3), and then receiving information about HIV prevention combined with sexual health (11.0, 95% CI 10.4–11.7). The lowest preference scores were for receiving information via a piece of paper or pamphlet (1.7, 95% CI 1.3–2.1), followed by receiving information via a website (2.0, 95% CI 1.6–2.4), and via a mobile phone application (2.9, 95% CI 2.3–3.5) (Fig. 2; Table 3). Receiving information from a doctor was almost eight times as preferred as receiving information via a piece of paper or pamphlet. 

**Table 3:** Preference scores for receiving HIV prevention education of full data and those excluded with fast completion times and low fit scores

Attribute	Level	Full survey data	Excluding participants with fast completion and low fit scores
		Preference score (95% CI)	Preference score (95% CI)
		*n* = 259	*n* = 238
Who			
	A nurse	10.6 (9.9–11.2)	10.7 (10.0-11.3)
	A doctor	13.4 (12.7–14.0)	13.5 (12.8–14.1)
	A counselor	8.5 (7.9-9.0)	8.4 (7.7-9.0)
Where			
	At a clinic visit	8.4 (7.8–8.9)	8.5 (7.9–9.1)
	At the hospital	8.5 (7.9-9.0)	8.5 (7.9–9.1)
	At a telemedicine or virtual visit	4.9 (4.2–5.6)	4.9 (4.2–5.6)
	At your own home	3.5 (3.0–4.0)	3.5 (2.9-4.0)
What			
	HIV prevention alone	5.1 (4.6–5.6)	5.0 (4.4–5.5)
	HIV prevention and sexual health	11.0 (10.4–11.7)	11.3 (10.6–12.0)
	HIV prevention and taking care of your health after you’ve had a baby	11.6 (10.9–12.3)	11.8 (11.0-12.5)
How			
	Website	2.0 (1.6–2.4)	1.7 (1.3–2.1)
	Mobile phone application	2.9 (2.3–3.5)	2.7 (2.1–3.3)
	Piece of paper or pamphlet	1.7 (1.3–2.1)	1.5 (1.2–1.9)
	Talk to you to find out what information you need	7.9 (7.2–8.6)	8.0 (7.3–8.8)

Abbreviations: HIV- Human immunodeficiency virus

Latent class analysis identified three classes of participant preferences for receiving HIV prevention information (Fig. 2; Table 2). Class One (*n* = 123, 47%) had highest preference scores related to *who* gave them information and for receiving information in person at either a clinic visit or at the hospital (referred to as “high touch” in subsequent discussion). Class Two (*n* = 91, 35%) had greatest preference for receiving HIV prevention information in combination with additional health information (referred to as “integrated information”). Class Three (*n* = 45, 17%) had greatest preferences for receiving information utilizing technology (e.g., via mobile phone application, telemedicine visit) (referred to as “high technology”).

In bivariate analyses, age, race and ethnicity did not differ between the three classes. Sixty-six percent of participants in the high touch class had a high school degree or less relative to 38% in the integrated information class and 40% in the high technology class (*p* < 0.01). Classes also differed by the percentage who reported accessing HIV/STI services prior to pregnancy, with only 29% having accessed services in the high touch class relative to 36% in the integrated information class and 49% in the high technology class. A lower percentage of participants in the high technology class agreed with the statements, “I worry about getting HIV,”, “It’s easy for me to get HIV and STI prevention services,” and “I want to learn more about ways to prevent HIV” relative to other classes.

In TURF analysis, reach was 90% with a portfolio that included (1) who: a doctor providing the information, (2) where: the information being provided at a telemedicine visit, (3) what: information provided is about HIV prevention combined with taking care of their health after having a baby, and (4) how: information is provided verbally. When the *what* in the above portfolio changed to information about HIV prevention combined with sexual health information and all other items ( who, where, how) remained the same, reach remained at 90%.

### Preferences for PrEP Messaging

**Fig. 3:**
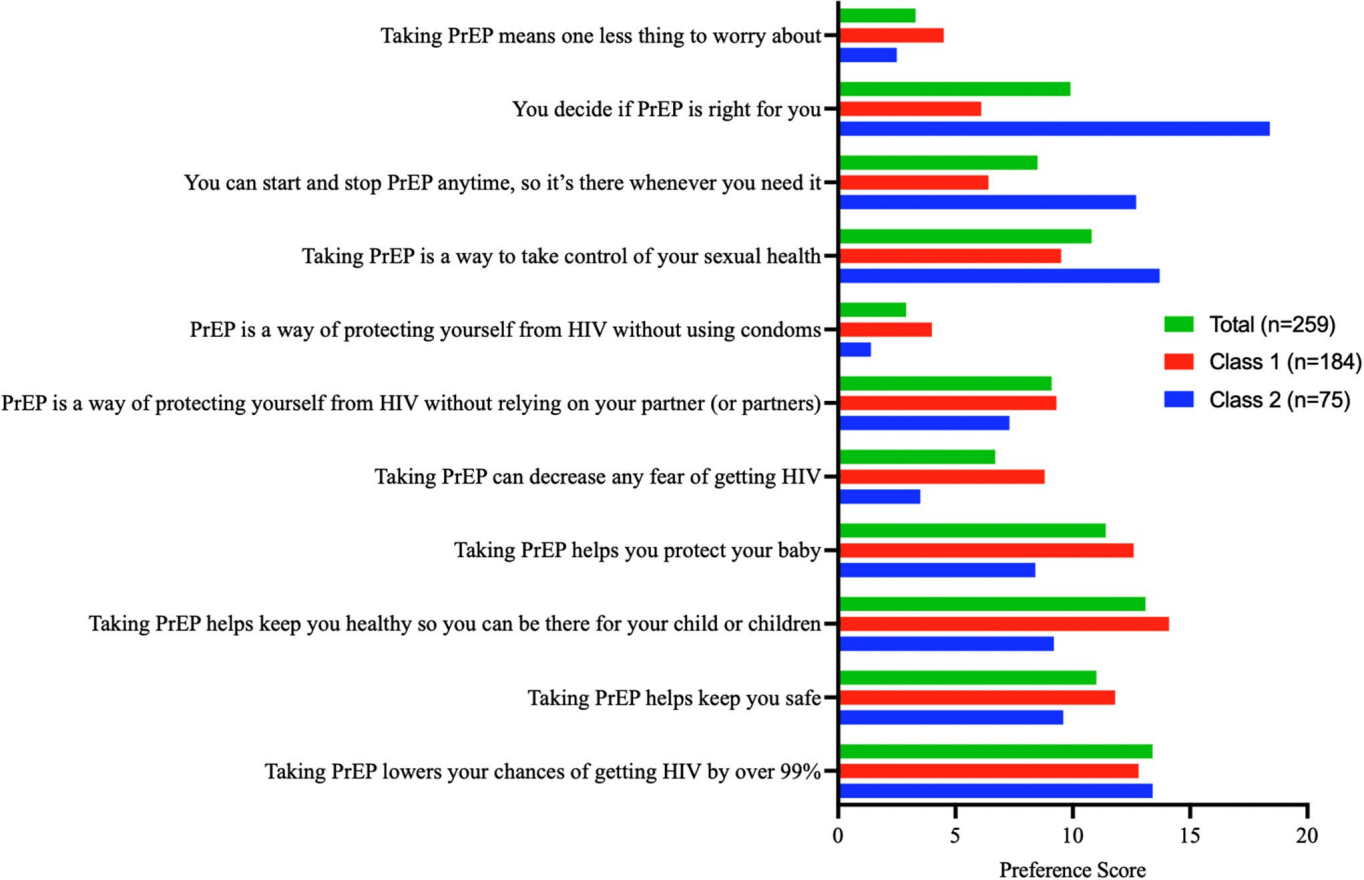
Preference scores from best-worst scaling exercise of messages related to HIV pre-exposure prophylaxis (PrEP) by total study population (*n* = 259) and by two latent classes

When asked about messaging related to PrEP, highest preference scores were related to themes of effectiveness, “Taking PrEP lowers your chances of getting HIV by over 99%” (13.4, 95% CI 12.7–14.2); motherhood, “Taking PrEP helps keep you healthy so you can be there for your child or children” (13.1%, 95% CI 12.4–13.8) and “Taking PrEP helps you protect your baby” (11.4, 95% CI 10.8–11.9); and safety and autonomy, “Taking PrEP helps keep you safe” (11.0, 95% CI 10.6–11.5) and “Taking PrEP is a way to take control of your sexual health” (10.8, 95% CI 10.1–11.4) (Fig. 3; Table 4). Participants had lower preference scores for messaging related to protection without condom use, “PrEP is a way of protecting yourself from HIV without using condoms” (2.9, 95% CI 2.5–3.3); and fear or worry, “Taking PrEP can decrease any fear of getting HIV,” (6.7, 95% CI 6.2–7.2) and “Taking PrEP means one less thing to worry about” (3.3, 95% CI 3.0-3.6). The message of “Taking PrEP lowers your chances of getting HIV by over 99%” was four and a half times more preferred than the message of “PrEP is a way of protecting yourself from HIV without using condoms.”

**Table 4:** Preference scores for PrEP messaging of full data and those excluded with fast completion times and low fit scores

PrEP message	Full survey data	Excluding participants with fast completion and low fit scores
	Preference score (95% CI)	Preference score (95% CI)
	*n* = 259	*n* = 188
Taking PrEP lowers your chances of getting HIV by over 99%	13.6 (12.7–14.2)	14.6 (13.5–15.6)
Taking PrEP helps keep you safe	11.0 (10.6–11.5)	11.0 (10.3–11.6)
Taking PrEP helps keep you healthy so you can be there for your child or children	13.1 (12.4–13.8)	13.5 (12.6–14.4)
Taking PrEP helps you protect your baby	11.4 (10.8–11.9)	11.8 (11.0-12.6)
Taking PrEP can decrease any fear of getting HIV	6.7 (6.2–7.2)	5.7 (5.0-6.3)
PrEP is a way of protecting yourself from HIV without relying on your partner (or partners)	9.1 (8.4–9.8)	9.2 (8.2–10.1)
PrEP is a way of protecting yourself from HIV without using condoms	2.9 (2.5–3.3)	1.5 (1.2–1.9)
Taking PrEP is a way to take control of your sexual health	10.8 (10.1–11.4)	11.5 (10.6–12.4)
You can start and stop PrEP anytime, so it’s there whenever you need it	8.5 (7.8–9.2)	8.5 (7.5–9.4)
You decide if PrEP is right for you	9.9 (9.1–10.7)	10.5 (9.4–11.7)
Taking PrEP means one less thing to worry about	3.3 (3.0-3.6)	2.2 (1.9–2.5)

Abbreviations: PrEP- pre-exposure prophylaxis; HIV- Human immunodeficiency virus

In latent class analysis of message preferences, two classes were identified (Fig. 3; Table 2). Both classes had greater preferences for messages related to effectiveness, however Class One (*n* = 184, 71%) had higher preference scores on messaging related to motherhood and safety whereas Class Two (*n* = 75, 29%) had higher preference scores on messaging related to control and autonomy. A significantly higher percentage of participants in the class preferring messaging related to motherhood and safety had not accessed HIV or STI services prior to pregnancy than those in the class who preferred messaging related to control or autonomy (71% vs. 52%, *p* < 0.01). Additionally, those participants in the class preferring messaging related to control and autonomy were significantly less likely to agree that creation of a sexual health plan would be overwhelming compared to those in the other class (11% vs. 25%, *p* = 0.01).

In TURF analysis, reach of the two messages, “Taking PrEP lowers your chances of getting HIV by over 99%,” and “Taking PrEP helps keep you healthy so you can be there for your child or children” was 64%. When three item portfolios were evaluated, the addition of the message “*You* decide if PrEP is right for you,” demonstrated a cumulative reach of 72%.

## Discussion

In this study, postpartum CGW’s knowledge of PrEP was low, with 53% having never previously heard of PrEP. However, most participants were interested in learning more, and 45% responded that they might or would use PrEP in the future. Latent class analyses of BWS preference scores demonstrated heterogeneity of preferences for delivery of HIV prevention information and PrEP messaging suggesting the potential benefit for patient-centered, differentiated delivery of HIV prevention education and services to support acceptability and receptivity in postpartum CGW.

Most participants in this study had no prior knowledge of PrEP and only 4% knew a lot about PrEP demonstrating that a substantial knowledge gap remains. In a larger survey among Black and Latina CGW living in NYC in 2018, only 45% had heard of PrEP [1]. Access to HIV and STI services prior to pregnancy was positively associated with knowledge of PrEP, however 65% of participants had not accessed these services. This underscores the critical opportunity that prenatal and postpartum care can provide to support the sexual health of CGW and provide PrEP education.

While most participants were in the high touch class and had a greater preference for receiving information in person (e.g., at a clinic visit or during the postpartum hospital stay) from a doctor or nurse, a smaller group of participants preferred receiving information utilizing technology. The emergence of both a high touch and high technology class suggests that delivery of HIV prevention information may need to be individualized based on patient’s preferences to maximize receptivity. While telehealth models for PrEP care have demonstrated acceptability among users [18], delivery of HIV prevention services solely by telehealth may not be the preferred method of service delivery among all CGW. However, having a telehealth option for receiving PrEP care may increase reach in CGW, as evidenced by this option appearing in the portfolio of services with the highest reach in TURF analysis. While in the high technology class the highest preference score for how to receive information was a mobile application, participants also had a higher relative preference score for receiving information verbally.

More participants in the high technology class had accessed HIV and STI services outside of pregnancy but were also less likely to agree that it was easy for them to access HIV prevention services than other classes. The high technology class may have a greater understanding of logistical and health system barriers to receiving HIV and STI services (e.g., long wait times, time traveling to an appointment, etc.) and these experiences may inform their desire to utilize technology to receive health information to support overcoming logistical barriers. Additionally, participants in the high technology class had a lower percentage who expressed worry about potential HIV acquisition and interest in learning more about HIV prevention strategies relative to the other classes, suggesting that this class may have a lower perceived risk of HIV than other classes. This low perceived risk could potentially facilitate their preference for receiving information in a high technology setting. Further studies are needed to elucidate factors influencing a patient’s preference for receiving technology-based services to better understand who may benefit most.

Overall, participants had a greater preference for receiving HIV prevention information integrated with sexual or maternal health care information, suggesting patients’ desire for health education and empowerment in their health decisions. In addition, participants' preference for integrated HIV prevention information may be driven by stigma; providing HIV prevention education as a part of comprehensive postpartum health education may be destigmatizing while the separation of HIV prevention services may perpetuate stigma [19]. Other studies have demonstrated that positive PrEP social norms are associated with interest in using PrEP and suggest that interventions that normalize PrEP discussions may support positive PrEP social norms and facilitate PrEP uptake [20].

Participants also preferred PrEP messaging related to effectiveness. While this remained a preferred message in both classes in latent class analysis, one class of participants had a greater preference for messages related to motherhood and safety, and the other class had a greater preference for messages related to control and autonomy. Participants’ preference for messages related to their role as a mother suggests that postpartum patients may be particularly receptive to PrEP education during this period. However, while motherhood may motivate prioritization of sexual health and PrEP uptake, healthcare should be comprehensive and recognize the patients as more than just mothers. Interestingly, messaging related to fear, worry or protection had the lowest preference scores. These preferences in this population may be a result of low actual or perceived risk of HIV acquisition, as only 40% of our participants agreed that they worry about acquiring HIV. Framing discussions about PrEP as empowering and prioritizing health both independently and in the context of motherhood may maximize receptivity. In this study population, motivation for PrEP use may be less about fear or worry over acquisition of HIV and more about prioritization and control in supporting individual health. Multiple messages may need to be delivered to effectively reach all patients who may benefit.

This study has some limitations. First, all participants were recruited from a single academic, urban hospital among participants able to complete the survey in English or Spanish. Thus, results may not be generalizable to all postpartum CGW in the US but provides valuable information in an area of high HIV prevalence. While this study focused on CGW, all individuals with uteruses giving birth need access to comprehensive postpartum care. Additionally, when comparing demographics from study participants to eligible patients on the postpartum unit there was a statistically significant difference in race, with more of the unit population identifying as a race other than those listed. While our sample may have an overrepresentation of individuals of Black or White race, data from the unit population was extracted from the EMR and survey data was self-reported. Patient race and ethnicity data in the EMR can be unreliable due to varying data collection and reporting practices [21]. Additionally, there is a potential for participants to have experienced survey fatigue which can result in inaccurate data. However, in the sensitivity analysis, results excluding participants with fast completion of the survey and those with low fit-scores resulted only in slight changes in the magnitude of preference scores. Family members were not asked to leave during survey completion potentially affecting participants’ responses. However, a study team member was present to observe survey completion and only one participant was excluded for involvement of someone else. The presence of partners would also be unlikely to influence preferences related to the delivery of information (i.e., if received from a doctor or nurse) but the presence of partners could potentially have affected attitudes towards and interest in PrEP. Due to HIV and PrEP stigma, we anticipate, if anything, that partner presence would have resulted in less favorable attitudes towards PrEP and more interest in learning about PrEP. Additionally, definitions of attribute levels were not provided to participants and were based on individual interpretation. For example, communication of information using a mobile phone application could be interpreted by one participant as a part of an existing healthcare communication application (e.g., Epic MyChart) or a new, separate application by another participant. Finally, our study included data from 259 participants. A larger sample size could have resulted in different classes and would have allowed for further segmentation of analyses.

The study findings support that routine obstetrical care provides an opportunity for delivery of comprehensive sexual health education that includes information about HIV prevention services and PrEP. The heterogeneity in preferences for receiving information at the patient level suggests the need for patient-centered, differentiated service delivery to support maximum receptivity and interest, for example a choice between an in-person or telemedicine visit to provide HIV prevention education. Additionally, participants’ preferences underscore the value they place on receiving HIV prevention information and services as a part of comprehensive maternal health education and care and provides valuable input about how information can be delivered to maximize acceptability. The focus of this study was on overcoming patient level barriers, however additional barriers exist at the provider and health systems level which must be overcome to support successful integration of HIV prevention services into postpartum care.

## Electronic Supplementary Material

Below is the link to the electronic supplementary material.

Supplementary Material 1

## Data Availability

Due to the sensitive nature of the research, supportive data is not available.
