# Supplementary material for: Experiences, Attitudes and Preferences of Postpartum Cisgender Women for HIV Prevention and Pre-Exposure Prophylaxis Education during Routine Postpartum Care
Source: AIDS Behav. 2025 Mar 10;29(6):1973–83. doi: 10.1007/s10461-025-04663-5 (PMC12074870; doi:10.1007/s10461-025-04663-5)
Supplement: Supplementary file 1 — Supplementary Material 1 [file 10461_2025_4663_MOESM1_ESM.docx]

| **Supplemental Table 1. Characteristics of population surveyed and postpartum unit population over study period** | | | |
| --- | --- | --- | --- |
| **Characteristic** | **Survey population (n=259)** | **Unit population (n=902)** | **p** |
| Age^a^- mean (range) | 30 (19-46) | 30 (18-46) | 0.45 |
| Race^a^ |  |  | <0.001* |
| Black | 109 (46%) | 267 (32%) |  |
| White | 41 (17%) | 55 (7%) |  |
| Asian | 17 (7%) | 55 (7%) |  |
| Other | 71 (30%) | 464 (55%) |  |
| Ethnicity^a^ |  |  | 0.40 |
| Hispanic/Latina | 154 (60%) | 482 (57%)_ |  |
| Non-Hispanic/Latina | 104 (40%) | 368 (43%) |  |
| Primary Language^a^ |  |  | 0.21 |
| English | 223 (86%) | 744 (83%) |  |
| Spanish | 36 (14%) | 154 (17%) |  |

^a^ Denotes missing data. Survey population includes 255 with age, 238 with race, 258 with ethnicity; Unit population includes 841 with race, 850 with ethnicity, 898 with language.
